# Determination of Gnetol in Murine Biological Matrices by Liquid Chromatography–Tandem Mass Spectrometry (LC–MS/MS): Application in a Biodistribution Study

**DOI:** 10.3390/ijms262110358

**Published:** 2025-10-24

**Authors:** Boyu Liao, Hongrui Jin, Huan Chen, Yuxin Zhang, Xuexian Deng, Jingyi Yao, Na Li, Shaoshu Xu, Jingbo Wang, Mingming Gao, Xiaoying Zhang, Paul C. L. Ho, Hui Liu, Hai-Shu Lin

**Affiliations:** 1 School of Pharmacy, Shenzhen University Medical School, Shenzhen University, Shenzhen 518055, China; 2 College of Pharmacy, Shenzhen Technology University, Shenzhen 518118, China; 3 Department of Pharmacy, National University of Singapore, Singapore 119260, Singapore; 4 School of Pharmacy, Monash University Malaysia, Jalan Lagoon Selatan, Bandar Sunway, Subang Jaya 47500, Malaysia; 5 Quality and Standards Academy, Shenzhen Technology University, Shenzhen 518118, China

**Keywords:** gnetol, resveratrol, LC–MS/MS, nutraceutical, biodistribution

## Abstract

Gnetol (*trans*-2,3′,5′,6-tetrahydroxystilbene), a naturally occurring stilbene structurally related to resveratrol (*trans*-3,5,4′-trihydroxystilbene; RES), has been reported to possess multiple health-promoting activities. In order to support its potential nutraceutical application, a reliable chromatography–tandem mass spectrometry (LC–MS/MS) assay was developed and validated for the quantitative determination of gnetol in mouse plasma and tissue samples, using isotopically labeled RES-^13^C_6_ serving as the internal standard (IS). Electrospray ionization (ESI) was performed in negative mode, with multiple reaction monitoring (MRM) transitions *m*/*z* 243.2 → 175.0 for gnetol and *m*/*z* 233.1 → 191.0 for the IS. Chromatographic separation was achieved on a reversed-phase HPLC column using a 5-min gradient delivery of acetonitrile and 2 mM ammonium acetate at 0.5 mL/min and 40 °C. The linear calibration curve covered the concentration range of 5.0–1500 ng/mL, and the method validation confirmed its selectivity, accuracy, precision, stability, and dilution integrity. The developed method was subsequently applied to a biodistribution study in mice after oral administration of gnetol at 400 µmol/kg (equivalent to 97.7 mg/kg). Gnetol was rapidly absorbed and extensively distributed in key pharmacologically relevant organs. Despite its poor aqueous solubility, oral uptake was not significantly hindered. Collectively, these findings demonstrate that gnetol exhibits favorable absorption and tissue distribution profiles, supporting its promise as a candidate for nutraceutical development.

## 1. Introduction

**Figure 1:**
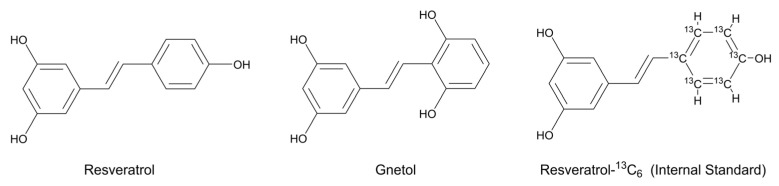
Chemical structures of resveratrol, gnetol and resveratrol-^13^C_6_ (internal standard).

Resveratrol (RES; *trans*-3,4′,5-trihydroxystilbene, Figure 1) is a plant-derived stilbene polyphenol that occurs abundantly in grapes, berries, peanuts, and red wine, as well as in several medicinal and edible plants [1,2,3,4]. During the past twenty years, RES has been extensively studied because of its diverse biological activities, which include antioxidant, anti-inflammatory, anti-diabetic, anti-obesity, and anti-carcinogenic properties, together with protective effects on the cardiovascular, hepatic, and nervous systems demonstrated in a wide array of experimental models [1,2,3,4]. More recently, clinical investigations have expanded to evaluate its potential therapeutic applications in conditions such as neurodegenerative diseases, cardiovascular disorders, malignancies, metabolic syndrome, non-alcoholic fatty liver disease, obesity, and osteoarthritis [3,4,5,6,7,8,9]. Owing to such pleiotropic actions, structurally related stilbenoids from dietary or herbal origins have also emerged as an area of growing scientific interest.

Gnetol (*trans*-2,3′,5′,6-tetrahydroxystilbene, Figure 1) is a naturally occurring stilbenoid and a close structural analog of RES, first isolated from *Gnetum ula* [10]. It has since been identified in several *Gnetum* species traditionally used in Oriental medicine [11,12,13,14,15,16,17], and more recently in dietary sources such as melinjo (*Gnetum gnemon*), a fruit widely consumed in Southeast Asia, suggesting that gnetol may constitute a natural dietary component in certain populations [11]. Over the past decade, growing pharmacological evidence has highlighted gnetol’s broad bioactivity profile. Similar to RES, it exhibits anti-cancer, anti-diabetic, anti-inflammatory, anti-obesity, cardioprotective, and hepatoprotective effects in preclinical models [11,18,19,20,21,22,23]. In addition, gnetol has been identified as a potent tyrosinase inhibitor, suggesting potential in cosmetic and dermatological applications such as skin-whitening and anti-hyperpigmentation therapies [24]. Preliminary data further demonstrate neuroprotective activity, implying possible utility in neurodegenerative disorders [17,23,25,26,27]. Notably, gnetol also inhibits the SARS-CoV-2 main protease, revealing promising antiviral potential [28]. Collectively, these findings establish gnetol not merely as a structural analog of RES but as a distinct bioactive stilbenoid with multifaceted therapeutic promise. Its presence in both medicinal plants and edible sources underscores the importance of continued investigation into its pharmacological properties and translational potential for preventive and therapeutic applications.

Accurate quantification of gnetol in biological matrices is crucial for elucidating its pharmacokinetic characteristics, tissue distribution, and bioavailability, which are fundamental to understanding its in vivo efficacy and safety. Given the growing interest in gnetol as a RES analog with promising health-promoting properties, a reliable analytical method is indispensable for supporting both preclinical evaluation and potential nutraceutical development. To date, only two pioneering studies have attempted to characterize the pharmacokinetic profile of gnetol in rats using high-performance liquid chromatography (HPLC) coupled with ultraviolet (UV) detection [11,12]. However, the relatively high lower limit of quantification (LLOQ) of 500 ng/mL limits the feasibility of conducting effective pharmacokinetic investigations, particularly those relevant to clinical or nutritional applications. Moreover, analytical methods for determining gnetol in tissue homogenates have not yet been reported. Therefore, in this study, we established and validated a sensitive and reliable liquid chromatography–tandem mass spectrometry (LC–MS/MS) method for quantifying gnetol in murine biological matrices. This method was subsequently applied to investigate the biodistribution of gnetol in mice after oral dosing. The findings are expected to provide valuable insights that support further pharmacological and nutraceutical exploration of gnetol and related RES analogs.

## 2. Results

### 2.1. Optimization of LC–MS/MS Parameters

**Figure 2:**
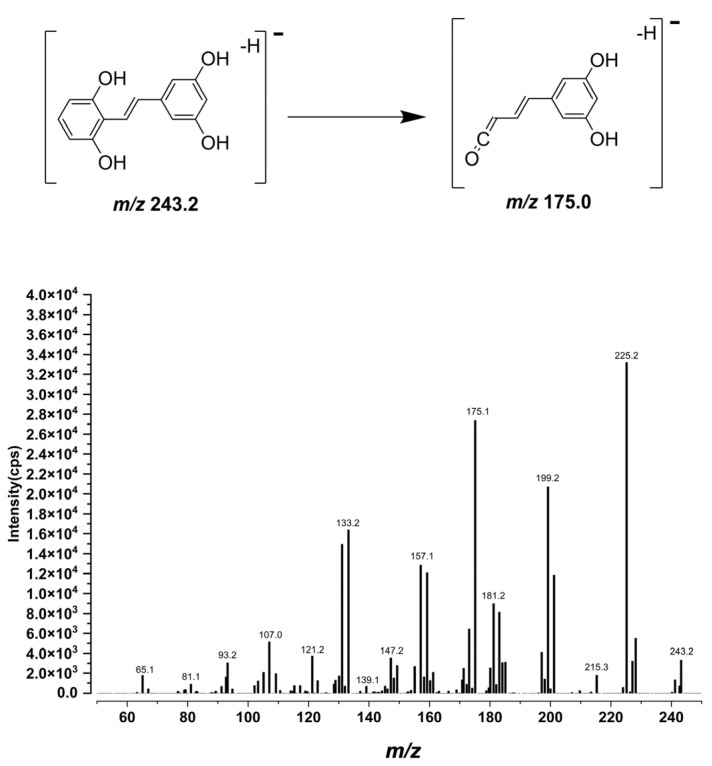
MS/MS spectra of gnetol and proposed fragmentation pattern of *m*/*z* 243.2 → 175.0 transition. Gnetol (1000 ng/mL) was directly infused into the mass spectrometer to obtain the MS/MS spectra.

During method development, mass spectra of gnetol were acquired in both electrospray ionization (ESI) polarities. The deprotonated ion gave a markedly stronger signal than the protonated ion; therefore, multiple reaction monitoring (MRM) analyses were performed in negative ESI mode. After collision-energy optimization, the quantifier MRM transition *m*/*z* 243.2 → 175.0 was selected as it provided the best balance of selectivity and sensitivity. The MS/MS spectrum and the proposed fragmentation pathway are shown in Figure 2. The internal standard (IS) was monitored using the MRM transition *m*/*z* 233.1 → 191.0, as in our previous studies [29,30].

Chromatographic parameters—including mobile-phase composition and the gradient program—were optimized to produce sharp, symmetrical peaks for gnetol and the IS while keeping the total run time within 5 min.

### 2.2. Assay Validation

This LC-MS/MS method was validated by the examination of its selectivity, sensitivity, precision, accuracy, matrix effect, and stability profiles of gnetol.

**Figure 3:**
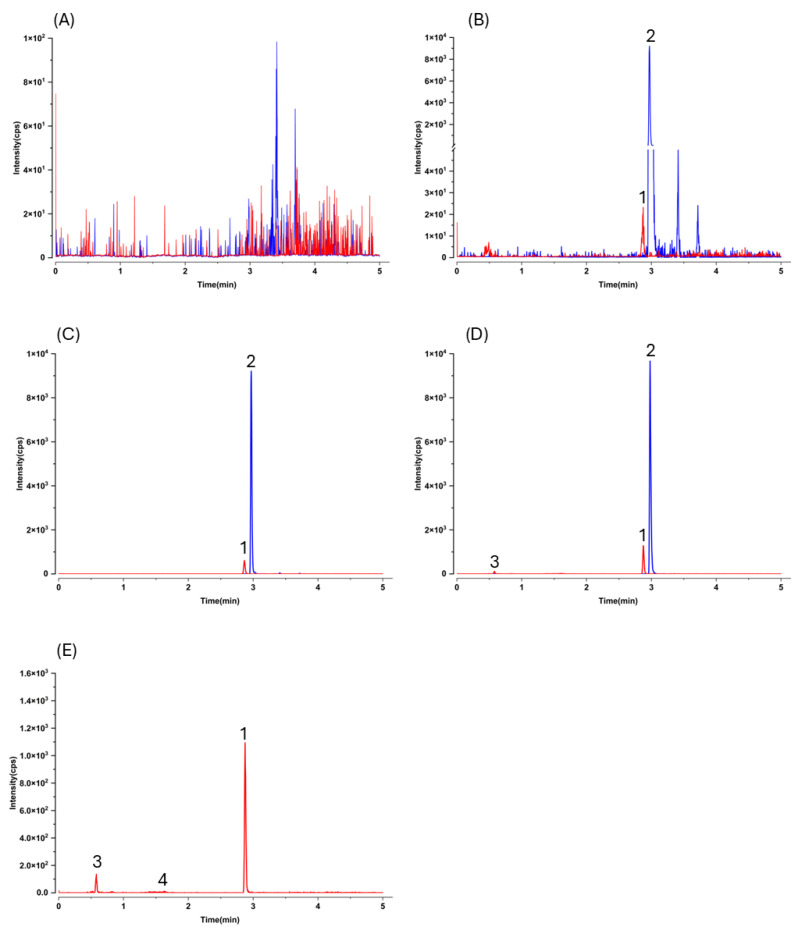
Representative LC–MS/MS chromatograms of mouse plasma. Multiple reaction monitoring transitions of *m*/*z* 243.2 → 175.0 (red) and *m*/*z* 233.1 → 191.0 (blue) were used for gnetol and the internal standard (resveratrol-^13^C_6_), respectively. (**A**) Blank mouse plasma; (**B**) blank plasma spiked with gnetol at lower limit of quantification (5.0 ng/mL) and internal standard (1500 ng/mL); (**C**) blank plasma spiked with gnetol (300 ng/mL) and internal standard (1500 ng/mL); (**D**) plasma obtained 10 min after oral administration of gnetol at 400 µmol/kg (97.7 mg/kg) in the presence of internal standard; and (**E**) plasma obtained 10 min after oral administration of gnetol at 400 µmol/kg without internal standard. Peaks: 1, gnetol; 2, internal standard; 3 & 4, unidentified metabolites.

No interfering peaks were observed at the retention times of gnetol or the IS in blank mouse plasma (n ≥ 6) monitored at the MRM transitions of *m*/*z* 243.2 → 175.0 (gnetol) and *m*/*z* 233.1 → 191.0 (IS). Representative MRM chromatograms of blank mouse plasma, blank plasma spiked with gnetol at LLOQ (5.0 ng/mL) and IS (1500 ng/mL), blank plasma spiked with gnetol (300 ng/mL) and IS are shown in Figure 3A, Figure 3B and Figure 3C, respectively. Under the present chromatographic conditions, gnetol (peak 1) and the IS (peak 2) eluted at ~2.8 and ~2.9 min, respectively. Representative chromatograms of plasma collected after oral administration of gnetol at 400 µmol/kg (97.7 mg/kg) are shown in Figure 3D (with IS) and Figure 3E (without IS). Unidentified peaks eluting at approximately 0.6 min (peak 3) and 1.6 min (peak 4) were also observed. These peaks are presumably metabolites, as they were detected only in post-dosing samples. Importantly, none of these metabolites interfered with the detection of either gnetol or the IS.

**Figure 4:**
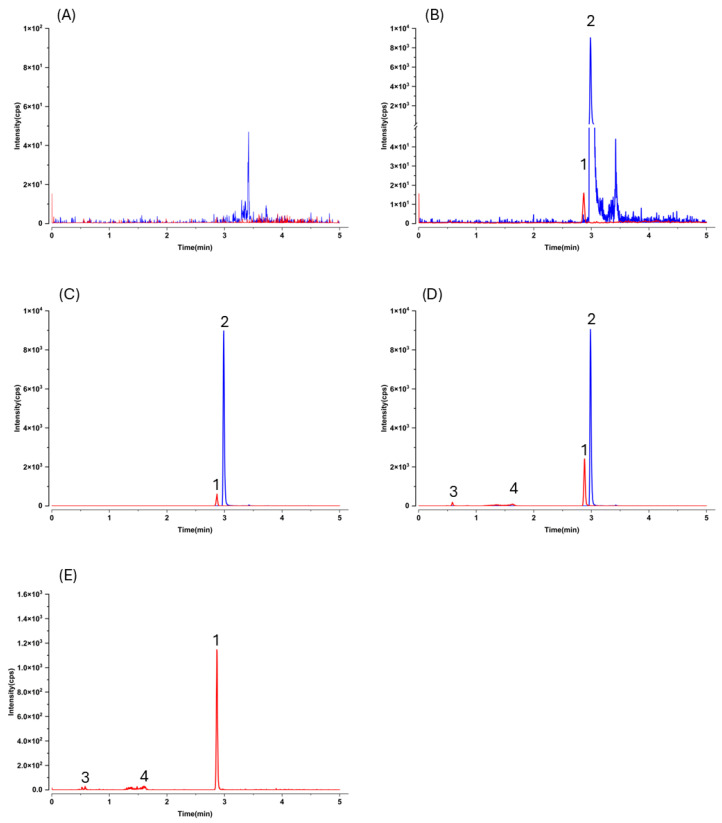
Representative LC–MS/MS chromatograms of mouse hepatic homogenate samples. Multiple reaction monitoring transitions of *m*/*z* 243.2 → 175.0 (red) and *m*/*z* 233.1 → 191.0 (blue) were used for gnetol and the internal standard (resveratrol-^13^C_6_), respectively. (**A**) Blank mouse hepatic homogenate; (**B**) blank hepatic homogenate spiked with gnetol at lower limit of quantification (5.0 ng/mL in homogenate, corresponding to 30 ng/g in tissue) and internal standard (1500 ng/mL); (**C**) blank homogenate spiked with gnetol (400 ng/mL in homogenate, corresponding to 2400 ng/mL in tissue) and internal standard (1500 ng/mL in homogenate); (**D**) hepatic sample obtained 10 min after oral administration of gnetol at 400 µmol/kg (97.7 mg/kg) in the presence of internal standard; and (**E**) hepatic sample collected 10 min after oral administration of gnetol at 400 µmol/kg without internal standard. Peaks: 1, gnetol; 2, internal standard; 3 and 4, unidentified metabolites.

Because liver homogenate is widely regarded as one of the most challenging matrices for LC–MS/MS, owing to its complexity and pronounced matrix effects, we validated the method in mouse liver homogenate to demonstrate its suitability for quantifying analytes in tissue homogenates. Representative MRM chromatograms of mouse liver homogenate are shown in Figure 4. Selectivity was again confirmed.

The lower limit of quantification (LLOQ) for this LC–MS/MS method was determined to be 5.0 ng/mL in both plasma and hepatic homogenate (corresponding to 30 ng/g in tissue). At this concentration, the signal-to-noise ratio was greater than 5:1 (Figure 3B and Figure 4B). The calibration curves are presented in Table 1.

The accuracy and precision of the LC–MS/MS method were evaluated using quality control (QC) samples (5.0, 15.0, 90.0, 600, and 1200 ng/mL) prepared in plasma and hepatic homogenate. Intraday and inter-day analyses were conducted in plasma and liver homogenate (Table 2). The assay demonstrated good accuracy and precision, with mean accuracies within 100 ± 15% and relative standard deviation (RSD) not exceeding 15% across all QC levels.

The absolute recovery (%) and matrix effect were evaluated using plasma and hepatic homogenate as matrices. The extraction efficiency was high, with the mean absolute recoveries ranging from 100 ± 15%, and the RSD staying below 15% across all tested levels (Table 3). Furthermore, the matrix effect was insignificant, with IS-normalized matrix factors remaining within 1.00 ± 0.15 and RSD under 10% (Table 3).

The stability profiles of gnetol and IS stock solutions under our handling conditions were examined and confirmed (Table 4).

In bioanalytical studies, maintaining the stability of an analyte within biological matrices is essential to guarantee precise and dependable quantification. If instability occurs, the compound may degrade or undergo transformation during sample handling, storage, or preparation, which can undermine data reliability and the overall validity of the study. For this reason, the stability of gnetol in plasma was carefully investigated under different storage conditions (Table 5). Evaluating its stability in intact tissues is inherently difficult, since the native microenvironment cannot be faithfully reproduced. Therefore, only short-term stability (6 h on ice) and post-preparative stability was examined in tissue homogenates (Table 5). Under all experimental conditions, gnetol demonstrated satisfactory stability.

The dilution integrity was confirmed (Table 6), as the mean recoveries were all within 100 ± 15% with RSD values not exceeding 15%.

In the present study, we successfully developed and validated a robust LC–MS/MS method for the quantitative determination of gnetol in biological matrices. This validated assay establishes a solid analytical foundation, enabling systematic and comprehensive biodistribution studies of gnetol in mice to be conducted with high accuracy and reliability.

### 2.3. Application to Biodistribution Study

**Figure 5:**
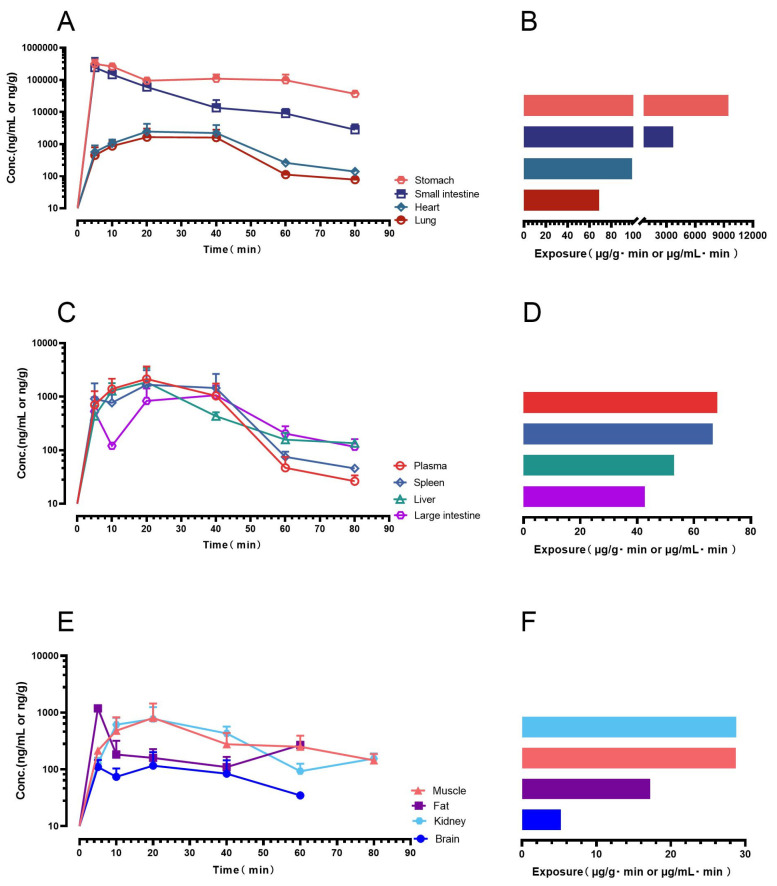
Biodistribution profiles and exposures of gnetol in mice following single oral administration at 400 μmol/kg (97.7 mg/kg). Symbol reprents mean value while error bar represents SD (*n* = 3 or 4). (**A**) concentation–time profiles & (**B**) exposure data (AUC) of gnetol in stomach (coral red), small intestine (navy blue), heart (dark cyan) and lung (brick red); (**C**) concentation–time profiles & (**D**) AUC of gnetol in plasma (scarlet), spleen (royal blue), liver (turquoise) and large intestine (bright purple); (**E**) concentation–time profiles & (**F**) AUC of gnetol in kidney (azure), muscle (salmon pink), fat (royal purple) and brain (electric blue).

Following oral administration of gnetol at a dose of 400 μmol/kg (corresponding to 97.7 mg/kg), groups of three to four mice (*n* = 3–4) were humanely euthanized at each scheduled sampling point (5, 10, 20, 40, 60, and 80 min). At every interval, plasma as well as representative tissue samples were promptly collected under controlled conditions to minimize degradation. The concentrations of gnetol in these matrices were subsequently determined using the LC–MS/MS method developed in this study. On the basis of the mean concentrations obtained at each time point, exposure levels expressed as the area under the concentration–time curve (AUC) were calculated for plasma and for each biological matrix. These quantitative results allowed the construction of detailed concentration–time profiles, from which the biodistribution and exposure characteristics of gnetol could be clearly visualized, as shown in Figure 5.

Gnetol exhibited remarkably rapid gastrointestinal absorption, being detected in the systemic circulation and multiple vital organs at relatively high concentrations as early as 5 min after oral administration. The absence of a discernible lag phase suggests efficient gastrointestinal permeability together with favorable physicochemical properties that promote oral uptake. The exposure profile followed the order: stomach > small intestine > heart > lung > plasma > spleen > liver > large intestine > kidney > muscle > fat > brain. Of particular note, gnetol was able to distribute into both lipophilic adipose tissue and the brain. Its early presence in the brain provides strong evidence of blood–brain barrier penetration, a critical property for agents with potential neuroprotective or neuromodulatory activities. Taken together, these findings indicate that a single oral dose of gnetol leads to rapid and extensive systemic distribution, ensuring its bioavailability in plasma and in most major pharmacological target organs.

## 3. Discussion

As expected, LC–MS/MS afforded markedly higher sensitivity and selectivity than HPLC–UV, but this advantage comes with an analytical caveat typical for pharmacokinetic work: without prior knowledge of metabolite structures, MRM transitions optimized for the parent may not capture conjugated species. Indeed, after oral dosing we observed additional early-eluting peaks that shared the gnetol transition (*m*/*z* 243.2 → 175.0) in both plasma and liver chromatograms (Figure 3D,E and Figure 4D,E). These signals most plausibly arise from phase II conjugates (e.g., glucuronides or sulfates) that undergo in-source cleavage to regenerate the aglycone and thus appear at the parental MRM. Such behavior has been reported for multiple phytostilbenes, including RES, pinosylvin, pinostilbene, isorhapontigenin (ISO), desoxyrhapontigenin, and *trans*-4,4′-dihydroxystilbene [29,30,31,32,33,34]. Consistent with this interpretation, gnetol glucuronidation has been documented in rats [11,12,35]. Nevertheless, definitive assignment will require authentic standards; enzymatic hydrolysis can only yield coarse totals and cannot resolve individual conjugate species. Given structural considerations and precedents with RES and piceatannol (PIC), sulfation is also likely [36], but dedicated metabolite profiling was beyond the present scope.

Despite its limited aqueous solubility, gnetol showed appreciable oral absorption (Figure 5), aligning with observations for other hydroxylated stilbenes—including RES, oxyresveratrol, PIC, rhapontigenin, and ISO [33,34,36]. We previously proposed an empirical principle that stilbenes bearing three or more hydroxyl groups can nonetheless achieve meaningful oral uptake irrespective of poor solubility [37], and gnetol conforms to this pattern. Accordingly, solubility-only strategies—although popular for other polyphenols [38,39,40,41]—may offer limited incremental value for gnetol. By contrast, formulations designed to prolong exposure (e.g., sustained-release systems) may better maintain effective plasma levels, extend tissue residence, and ultimately enhance nutraceutical efficacy.

Orally administered gnetol was rapidly absorbed and broadly distributed. Peak concentrations occurred in the gastrointestinal tract (stomach, small intestine), with substantial systemic exposure in the circulation, heart, and lung; moderate levels were observed in the liver, spleen, and large intestine, and measurable amounts in kidney, adipose tissue, and skeletal muscle. Although brain levels were low, gnetol crossed the blood–brain barrier to a detectable extent. This profile has clear translational implications. High gastrointestinal exposure supports a rationale for local mucosal actions—reinforcing barrier integrity and mitigating oxidative/inflammatory stress—relevant to gastritis, ulcer susceptibility, and mild inflammatory bowel conditions. Systemic distribution to vascular and pulmonary tissues aligns with cardiopulmonary applications, paralleling the endothelial and vascular benefits widely reported for RES [1,2,3,4]. The combination of moderate hepatic exposure and discernible adipose accumulation is consistent with metabolic utility, given the central roles of hepatic oxidative stress and adipose inflammation in insulin resistance and fatty liver disease. Corroborating evidence from the related stilbene PIC—a natural RES analog and human RES metabolite [42]—shows reduced weight gain/adiposity with down-regulation of C/EBPα and PPARγ in obese mice [43,44], improved glucose tolerance in rodents [45], and improved insulin sensitivity with lowered blood pressure/heart rate in an 8-week human RCT of passion-fruit–seed extract standardized to PIC [46]. Together, these data reinforce the cardiometabolic potential of stilbenes, gnetol included. Notably, the spleen accumulation observed here—also reported for ISO [37]—raises the possibility of lymphatic involvement in absorption; if confirmed, gnetol’s scope may extend to conditions with lymphatic or immune components. Finally, consistent with RES, oxyresveratrol (OXY), and ISO [37,47,48,49], only modest brain exposure was observed, supporting an empirical structure–activity relationship in which stilbenes bearing ≥3 hydroxyl groups exhibit limited central nervous system penetration; accordingly, the hydroxylation pattern may serve as a structural predictor of brain pharmacokinetics. Notably, innovative oral formulations and favorable drug–drug interactions have been shown to enhance the brain bioavailability of RES and OXY in preclinical studies [47,50,51], pointing to promising directions for future investigation.

Limitations of this study include its single-dose, single-species design; the absence of dose–exposure proportionality and tissue time courses; and the lack of characterization of phase II metabolism (glucuronidation/sulfation). Future work should incorporate comprehensive metabolite profiling, long-term safety evaluation, and assessment of sex- and age-dependent effects, and should benchmark gnetol against RES, OXY, PIC, and ISO to refine structure–activity relationships and inform rational combination strategies for metabolic and inflammatory indications.

## 4. Materials and Methods

### 4.1. Special Precautions

All experimental operations with stilbenes were performed under reduced lighting conditions to minimize the risk of light-induced isomerization [37].

### 4.2. Chemicals and Reagents

Gnetol (*trans*-2,3′,5′,6-tetrahydroxystilbene; Figure 1; purity: >97.0%) was purchased from Tokyo Chemical Industry (Tokyo, Japan). Resveratrol-^13^C_6_ (*trans*-3,5,4′-trihydroxystilbene-^13^C_6_; Figure 1; purity: >95%), an isotopically labeled analogue of resveratrol with a mass shift of +6 Da, was obtained from Toronto Research Chemicals (Toronto, ON, Canada) and used as the internal standard. All additional reagents and chemicals employed in the experiments were of no less than analytical grade. High-purity water (18.2 MΩ·cm at 25 °C) together with chromatographic grade acetonitrile and methanol were consistently used during the analyses. Mouse blank plasma was sourced from Sbjbio (Nanjing, China).

### 4.3. Liquid Chromatography—Tandem Mass Spectrometry (LC–MS/MS)

All liquid chromatography–tandem mass spectrometry (LC–MS/MS) analysis was was conducted on an Agilent 6475A triple quadrupole mass spectrometer equipped with a TurboIonSpray probe, interfaced to an Agilent 1290 Infinity II UHPLC system (Agilent Technologies, Santa Clara, CA, USA).

All biological samples were analyzed on a reversed-phase HPLC column (Agilent, Poroshell 120 EC-C18; 3.0 × 75 mm i.d., 2.7 μm), which was protected by a guard column (Agilent, Poroshell 120 EC-C18; 5 × 3.0 mm, 2.7 μm). Chromatographic separation was carried out at 40 °C using a 5-min gradient elution with a mixture of acetonitrile and ammonium acetate (2 mM) at a flow rate of 0.7 mL/min. The gradient schedule was as follows: (a) 0–2 min, acetonitrile 15%; (b) 2–2.5 min, acetonitrile 15 → 75%; (c) 2.5–3 min, acetonitrile 75 → 95%; (d) 3–4 min, acetonitrile 95%; (e) 4–4.5 min, acetonitrile 95 → 15%; (f) 4.5–5 min, acetonitrile 15%.

In the mass spectrometer, nitrogen served as the nebulizing, curtain, and collision gases. Source parameters for the ESI interface were optimized for the gnetol and the IS by instrument tuning in accordance with previously described procedures [29,30]. During the initial method development, mass spectra of gnetol were acquired in both ESI polarities. Because the deprotonated ion gave a markedly stronger signal than the protonated ion, MRM analyses were performed in negative ESI mode with a dwell time of 100 ms. The ESI source parameters are summarized in Table 7.

Compound-specific mass-spectrometric parameters on the Agilent system were first optimized using the instrument’s auto-optimization routine and then refined manually. After collision-energy optimization, the quantifier MRM transition *m*/*z* 243.2 → 175.0 was selected as it offered the best balance of selectivity and sensitivity. For gnetol, the fragmentor voltage, cell accelerator voltage, and collision energy were set to 130, 5, and 19 V, respectively. The IS was monitored at *m*/*z* 233.1 → 191.0, as in our previous studies [29,30], with the fragmentor voltage, CAV, and CE set to 127, 5, and 27 V, respectively.

### 4.4. Sample Preparation

Stock solutions of gnetol and resveratrol-^13^C_6_ (IS) were prepared in DMSO at 1 mg/mL, stored at −20 °C, and protected from light. Calibration standards and QC samples were generated by serially diluting the gnetol stock into pooled blank murine plasma or hepatic homogenate.

Plasma samples were prepared by protein precipitation with acetonitrile containing the IS (500 ng/mL). In brief, mouse plasma was mixed with acetonitrile at a 1:3 (*v*/*v*) ratio, vortexed for 20 s, and centrifuged at 1503× *g* for 10 min at 4 °C. The clarified supernatant was decanted into glass inserts seated in autosampler vials. The procedure, adapted from prior reports [29,30], requires only 25 μL of plasma per analysis.

For tissue analysis (brain, fat, heart, small intestine, large intestine, kidney, liver, lung, muscle, spleen, and stomach), approximately 35 mg of each specimen was weighed and finely chopped with scissors. Samples were then homogenized in 0.2% (*w*/*v*) L-ascorbic acid in 50% (*v*/*v*) methanol at a 1:5 (*w*/*v*; tissue:medium) ratio using a high-throughput homogenizer (Wonbio-L; Wonbio, Shanghai, China) for ≤3 cycles at 60 Hz, 60 s per cycle. Protein precipitation was performed by adding three volumes of acetonitrile containing the IS (500 ng/mL) to one volume of homogenate. After vigorous vortex-mixing, the mixtures were centrifuged at 1902× *g* for 10 min at 15 °C, and the clarified supernatants were carefully transferred to autosampler (HPLC) vials for analysis. This tissue-processing protocol was also applied in our recent biodistribution study [37].

When analyzing real samples collected in the biodistribution study, if the concentration of gnetol in a biological matrix exceeded the upper limit of quantification, the samples were diluted with the corresponding blank matrix (plasma: dilution factor of 6; tissue homogenate: 6 or 600) prior to sample preparation. During each analysis, 3 μL of supernatant was injected into the LC–MS/MS system.

### 4.5. Assay Validation

Method validation for this LC–MS/MS assay was performed in accordance with established guidelines, assessing selectivity, sensitivity, linearity, accuracy (analytical recovery), precision (intra- and inter-day), absolute recovery, and stability [52,53].

Selectivity was evaluated by contrasting chromatograms from blank mouse plasma or liver homogenate (*n* = 6 donors) with those from the corresponding matrices fortified with gnetol and the IS.

Sensitivity was expressed as the LLOQ, defined as the lowest concentration yielding a signal-to-noise ratio ≥ 5 while meeting acceptance criteria for accuracy (mean analytical recovery 80–120%) and precision (RSD ≤ 20%).

The peak-area ratio of gnetol to the IS was used as the analytical response. Calibration curves were generated by plotting response (*y*) versus gnetol concentration (*x*) and fitting with weighted least-squares linear regression (weighting 1/*x*^2^), as described previously [29,30]. Calibration standards at 5.0, 10, 30, 100, 300, 900, 1350, and 1500 ng/mL were used to construct the curve and assess linearity.

Within-day and between-day performance was assessed using QC samples at five levels (5, 15, 90, 600, and 1200 ng/mL). Accuracy was expressed as the measured concentration relative to the nominal value, and precision was evaluated as the RSD. Acceptance criteria were: at the LLOQ, mean analytical recovery 80–120% with RSD ≤ 20%; for all other levels, mean recovery 85–115% with RSD ≤ 15%.

Absolute recovery and matrix effects were characterized in two representative matrices—mouse plasma and liver homogenate. Absolute recovery was determined by comparing gnetol peak areas from matrix-spiked extracts with those from neat solutions at matched concentrations. Matrix effects were evaluated using blanks from six individual mice. For each matrix, the matrix factor was calculated as the peak area in post-extraction spiked matrix divided by the peak area in a neat solution for gnetol and for the IS; the IS-normalized matrix factor was obtained as matrix factor of gnetol/matrix factor of IS. A matrix effect was considered negligible when the RSD of the IS-normalized MF was <15%.

Stability was assessed under a range of storage and handling conditions. Stock solutions of gnetol and the IS were evaluated after storage on ice (0–4 °C) and at −20 °C. Plasma stability of gnetol was examined under short-term conditions (on ice for 6 h), long-term storage (−80 °C for 7 days), three freeze–thaw cycles, and post-preparative holding (autosampler at 4 °C for 24 h). Because stability is difficult to determine directly in intact tissues, only short-term stability in liver homogenate (on ice for 6 h) was assessed; for processed tissue extracts, post-preparative stability was likewise evaluated using liver homogenate as a representative matrix. Stability was considered acceptable when 85–115% of the initial gnetol concentration remained.

The dilution integrity was evaluated using a dilution factor of 6 for plasma and 6 or 600 for hepatic homogenate. A mean recovery within 100 ± 15% and a RSD not exceeding 15% confirmed acceptable dilution integrity.

### 4.6. Animals

All experimental procedures complied with the ARRIVE guidelines [54]. The protocol for study design and animal care was reviewed and approved by the Institutional Animal Ethics Committee of Shenzhen Technology University (approval number: SZTUDWLL2025025; date: 5 March 2025).

Male ICR mice (6–8 weeks of age) were obtained from Shenzhen Glorybay Biotech Co. (Shenzhen, China) and maintained under specific pathogen-free (SPF) conditions. Animals were provided with free access to standard chow and water throughout the acclimatization and dosing periods. Gnetol was formulated as an aqueous suspension consisting of 0.3% sodium carboxymethylcellulose and 0.2% L-ascorbic acid (final concentration: 9.75 mg/mL) and administered orally by gavage at a dose of 400 μmol/kg (equivalent to 97.7 mg/kg; administration volume: 10.0 mL/kg). This dose was selected to allow molar-equivalent comparison across stilbenes and to ensure quantifiable systemic exposure, as previous studies have demonstrated that both RES and OXY commonly exerted significant in vivo disease-modifying effects at 100 mg/kg (RES: 438 µmol/kg; OXY: 409 µmol/kg) [55,56].

At predetermined time points (5, 10, 20, 40, 60, and 80 min post-dose), groups of three to four animals were sacrificed by cervical dislocation. Blood was collected via cardiac puncture into heparinized tubes and centrifuged to separate plasma. Immediately thereafter, major organs and tissues—including brain, adipose tissue, heart, small and large intestine, kidney, liver, lung, skeletal muscle, spleen, and stomach—were excised. Harvested samples were gently rinsed with isotonic saline, blotted to remove residual fluid, and snap-frozen on dry ice. All biological materials were subsequently stored at −80 °C until LC-MS/MS analysis. The experimental design of this biodistribution study was adapted from our earlier work that investigated isorhapontigenin distribution [37].

### 4.7. Pharmacokinetic Analysis

Non-compartmental pharmacokinetic analysis was performed using WinNonlin (Standard Version 1.0; Scientific Consulting, Inc., Apex, NC, USA) [37]. Given the study design, three to four mice were sacrificed at each predetermined time point. Plasma and tissue exposures, expressed as the area under the concentration–time curve (AUC), were calculated based on the mean concentration values of individual mice at each time point [37].

### 4.8. Statistics

Unless otherwise specified, data are expressed as mean ± SD (n as indicated).

## 5. Conclusions

In this work, we developed and validated a reliable LC–MS/MS assay for quantifying gnetol in murine biological matrices. The method was subsequently applied to a mouse biodistribution study. Following oral administration, gnetol was rapidly absorbed and attained appreciable exposure across multiple pharmacologically relevant tissues. This combination of oral availability and broad tissue distribution supports advancing gnetol as a nutraceutical candidate and motivates follow-up studies on metabolite profiling, exposure-prolonging formulations, and translational evaluation.

## Figures and Tables

**Table 1:** Calibration curves of gnetol in plasma and hepatic homogenate.

Biological Matrices	Calibration Equation	Range (ng/mL)	*R* ^2^
Plasma	*y* = 0.15983*x* − 0.0002	5.0–1500	0.9931
Hepatic Homogenate	*y* = 0.12093*x* + 0.0002	5.0–1500	0.9981

**Table 2:** Accuracy and precision of gnetol in mouse plasma and hepatic homogenate *.

Type of Matrice	Nominal Concentration in Matrice (ng/mL)	Intra-Day			Inter-Day		
		Measured Concentration (ng/mL) (Mean ± SD)	Precision (RSD%)	Mean Accuracy (%)	Measured Concentration (ng/mL) (Mean ± SD)	Precision (RSD%)	Mean Accuracy (%)
Plasma	5.0	4.3 ± 0.4	8.7	86.0	4.9 ± 0.6	11.9	98.0
	15.0	14.1 ± 1.2	8.2	93.8	13.8 ± 1.3	9.5	92.0
	90.0	80.6 ± 7.8	9.7	89.6	78.5 ± 9.8	12.4	87.2
	600	548.8 ± 35.5	6.5	91.5	538.4 ± 60.6	11.3	89.7
	1200	1121.8 ± 71.1	6.3	93.5	1125.7 ± 111.7	9.9	93.8
Hepatic Homogenate	5.0	5.0 ± 0.7	13.4	100.3	5.0 ± 0.6	12.4	99.2
	15.0	15.9 ± 1.6	10.2	105.7	16.0 ± 1.2	7.8	106.4
	90.0	83.6 ± 9.8	11.7	92.8	86.0 ± 9.4	10.9	95.5
	600	629.2 ± 51.9	8.2	104.9	579.9 ± 58.8	10.1	96.7
	1200	1219.0 ± 102.2	8.4	101.6	1130.4 ± 105.7	9.3	94.2

* Intraday analyses were conducted in six replicates, whereas inter-day analyses were performed over three consecutive days, with six replicates per day.

**Table 3:** Absolute recovery (%) and internal standard normalized matrix factor *.

Matrice	Concentration (ng/mL)	Absolute Recovery (%)		Matrix Factor	
		Mean ± SD	RSD	Mean ± SD	RSD
Plasma	15.0	95.6 ± 6.0	6.3	1.1 ± 0.1	5.9
	600	92.1 ± 7.5	8.2	1.1 ± 0.0	4.1
	1200	102.8 ± 11.9	11.6	0.9 ± 0.0	2.7
Hepatic Homogenate	15.0	92.5 ± 1.7	1.8	1.1 ± 0.1	7.2
	600	109.9 ± 5.3	4.8	1.0 ± 0.0	4.2
	1200	117.6 ± 3.5	3.0	1.0 ± 0.1	5.0

* *n* = 6.

**Table 4:** Stability of stock solutions *.

Storage Condition	Concentration (mg/mL)	Remaining (%, Mean ± SD)
Gnetol stored at ice-cold temperature for 24 h	1.00	101.3 ± 5.3
Gnetol stored at −20 °C for 7 days	1.00	98.2 ± 3.9
Resveratrol-^13^C_6_ stored at ice-cold temperature for 24 h	1.00	98.2 ± 3.9
Resveratrol-^13^C_6_ stored at −20 °C for 7 days	1.00	100.5 ± 3.6

* *n* = 6.

**Table 5:** Stability of gnetol in biological matrices *.

Stability Tests	Plasma		Hepatic Homogenate	
	Remaining (%, Mean ± SD)		Remaining (%, Mean ± SD)	
	15.0 ng/mL	1200 ng/mL	15.0 ng/mL	1200 ng/mL
After three freeze and thaw cycles	103.2 ± 11.6	111.7 ± 2.2	-	-
Short-term stability (stored at ice-cold temperature for 6 h)	100.4 ± 7.3	105.9 ± 1.8	96.1 ± 11.3	109.5 ± 4.4
Post-preparative stability (samples kept in an autosampler at 4 °C for at least 24 h)	94.0 ± 7.7	103.2 ± 7.6	104.5 ± 4.5	88.9 ± 6.4
Long term-stability (−80 °C for 7 days)	97.2 ± 6.3	111.7 ± 2.5	-	-

* *n* = 6.

**Table 6:** Dilution integrity *.

Dilution Factor	Analytical Recovery (%, Mean ± SD)		
	Plasma	Hepatic Homogenate	
	7200 ng/mL	7200 ng/mL	720,000 ng/mL
6	91.2 ± 7.4	87.9 ± 3.8	-
600	-	-	85.8 ± 2.5

* *n* = 5.

**Table 7:** Key electrospray ionization settings.

Gas Temperature (°C)	280
Gas Flow (L/min)	13
Nebulizer (psi)	40
Capillary Voltage (V)	4000
Sheath Gas Temperature (°C)	310
Sheath Gas Flow (L/min)	10.5
Nozzle Voltage (V)	1400

## Data Availability

The data presented in this study are available on request from the corresponding authors.

## Abbreviations

The following abbreviations are used in this manuscript:

AUC	Area under the concentration–time curve
ESI	Electrospray ionization
HPLC	High-performance liquid chromatography
IS	Internal standard
ISO	Isorhapontigenin
LC–MS/MS	Liquid chromatography–tandem mass spectrometry
LLOQ	Lower limit of quantification
MRM	Multiple reaction monitoring
QC	Quality control
PIC	Piceatannol
RES	Resveratrol
RSD	Relative standard deviation
*R*^2^	Correlation coefficient
UV	Ultraviolet
